# A Reweighted Scheme to Improve the Representation of the Neural Autoregressive Distribution Estimator

**DOI:** 10.1155/2018/6401645

**Published:** 2018-12-23

**Authors:** Zheng Wang, Qingbiao Wu

**Affiliations:** School of Mathematical Sciences, Zhejiang University, HangZhou, Zhejiang, China

## Abstract

The neural autoregressive distribution estimator(NADE) is a competitive model for the task of density estimation in the field of machine learning. While NADE mainly focuses on the problem of estimating density, the ability for dealing with other tasks remains to be improved. In this paper, we introduce a simple and efficient reweighted scheme to modify the parameters of the learned NADE. We make use of the structure of NADE, and the weights are derived from the activations in the corresponding hidden layers. The experiments show that the features from unsupervised learning with our reweighted scheme would be more meaningful, and the performance of the initialization for neural networks has a significant improvement as well.

## 1. Introduction

Feature learning is one of the most important tasks in the field of machine learning. A meaningful feature representation could be the foundation of the other procedures. Among the various methods, the restricted Boltzmann machine (RBM), which is a powerful generative model, has shown its ability to learn useful representations from many different types of data [1, 2].

RBM models the higher-order correlations between dimensions of the input. It is often used as a feature extractor, or the building blocks of various deep models, for instance, deep belief nets. In the latter case, the learned representations are fed to another RBM in the higher layer, and the deep architecture often leads to better performance in many fields [3–5]. Its variants [6–8]also have the capability to deal with various kinds of tasks.

While RBM has lots of advantages, it is not suited for the problem of estimating distribution, in other words, estimating the joint probability of the observation. To estimate the joint probability of a given observation, a normalization constant must be computed, which is intractable even for a moderate size of input. To deal with the problem, some other ways must be used to approximate the normalization constant, for example, annealed importance sampling [9, 10], which is complex and computational costing.

The neural autoregressive distribution estimator (NADE) [11] is a powerful model for estimating the distribution of data, which is inspired by the mean-field procedure of RBM. Computing the joint probability under NADE can be done exactly and efficiently. NADE and its variants [12–17] have been shown to be state-of-the-art joint density models for a variety of datasets.

While NADE mainly focuses on the distribution of the data, it also can be regarded as an alternative model to extract features from data.

Reweight approaches have made a lot of achievements in the field of machine learning. In some models of ensemble learning, such as AdaBoost [18], the importance of each sample in dataset would be reweighted to achieve better results. In some deep generative models, reweight approaches have been proposed to adjust the importance weights for the procedure of importance sampling [19, 20]. With the reweight approaches, the estimation of the gradients would be more accurate.

In this paper, we deal with the feature learned by NADE and propose a novel method to improve the quality of the representation via a simple reweighted scheme of the weights learned by NADE. The proposed method remains the structure of the model, and the procedure of computation remains simple and tractable.

The remainder of the paper is structured as follows. In Section 2, we review the important architecture of RBM and NADE, which is the foundation of our method and experiments. In Section 3, we introduce and analyze the reweighted scheme to improve the quality of features learned by NADE. In Section 4, we present a similar method for the case of initialization. We provide the experimental evaluation and demonstrate the results in Section 5. Finally, we make a conclusion in Section 6.

## 2. Review of RBM and NADE

In this section, we review the basic RBM model and emphasize the relationship between RBM and NADE.

A restricted Boltzmann machine is a kind of Markov random field that contains one layer of visible units **v** ∈ {0,1}^*D*^ and one layer of hidden units **h** ∈ {0,1}^*H*^. The two layers are connected with each other, and there are no connections intralayer.

The energy of the state {**v**, **h**} is defined as

(1) $$\begin{array}{c} E\left(v,h;\theta \right)=-\overset{D}{\underset{i=1}{\sum }}b_{i}v_{i}-\overset{D}{\underset{i=1}{\sum }}\overset{H}{\underset{j=1}{\sum }}W_{ij}v_{i}h_{j}-\overset{H}{\underset{j=1}{\sum }}c_{j}h_{j}, \end{array}$$

where {*W*_*ij*_} are the connecting weights between layers and {*b*_*i*_, *c*_*j*_} are the biases of each layer.

The probability of a visible state is

(2) $$\begin{array}{c} p\left(v;\theta \right)=\frac{1}{Z\left(\theta \right)}\underset{h}{\sum }\exp\left(-E\left(v,h;\theta \right)\right), \end{array}$$

where *Z*(*θ*)=∑_**v**,**h**_exp(−*E*(**v**, **h**; *θ*)) is the normalization constant.

Due to the intractability of the normalization constant, RBM is less competitive in the task of estimating distribution.

For a given observation, the distribution can be written as

(3) $$\begin{array}{c} p\left(v\right)=\overset{D}{\underset{i=1}{\prod }}p\left(v_{i}\left|v_{<i}\right.\right), \end{array}$$

where **v**_<*i*_ denotes the subvector of the observation before the *i*-th dimension. To evaluate the conditional distribution *p*(*v*_*i*_|**v**_<*i*_), a factorial distribution *q*(*v*_*i*_, **v**_>*i*_, **h**|**v**_<*i*_) is used to approximate *p*(**v**_*i*_, **v**_>*i*_, **h**|**v**_<*i*_):

(4) $$\begin{array}{c} q\left(v_{i},v_{>i},h\left|v_{<i}\right.\right)=\mu_{i}{\left(i\right)}^{v_{i}}{\left(1-\mu_{i}\left(i\right)\right)}^{1-v_{i}},\\ \underset{j>i}{\prod }\mu_{j}{\left(i\right)}^{v_{j}}{\left(1-\mu_{j}\left(i\right)\right)}^{1-v_{j}},\\ \underset{k}{\prod }\tau_{k}{\left(i\right)}^{h_{k}}{\left(1-\tau_{k}\left(i\right)\right)}^{1-h_{k}}. \end{array}$$

The minimization of the KL divergence between these two distributions leads to two important equations:

(5) $$\begin{array}{c} \tau_{k}\left(i\right)=\text{sig}\left(c_{k}+\underset{j\geq i}{\sum }W_{kj}\mu_{j}\left(i\right)+\underset{j<i}{\sum }W_{kj}v_{j}\right),\\ \mu_{j}\left(i\right)=\text{sig}\left(b_{j}+\underset{k}{\sum }W_{kj}\tau_{k}\left(i\right)\right), \end{array}$$

where sig(*x*)=1/(1+exp(−*x*)) is the sigmoid function.

The main structure of NADE is inspired by the mean-field procedure [21], and results in the following equations:

(6) $$\begin{array}{c} p\left(v_{i}=1\left|v_{<i}\right.\right)=\text{sig}\left(b_{i}+{\left(V^{T}\right)}_{i,\cdot }h_{i}\right),\\ h_{i}=\text{sig}\left(c+W_{\cdot ,<i}v_{<i}\right), \end{array}$$

where (**V**^*T*^)_*i*,·_ represents the *i*-th row in the transpose of matrix **V** and **W**_·,<*i*_ represents the first *i*-1 columns of matrix **W**, which connects the input with the corresponding hidden layers.

These two equations indicate that NADE acts like a feed-forward neural network, and the training procedure of NADE can be cast into the same framework as the common neural network while the cost function is the average negative log-likelihood of the training set. The gradient of the cost function with respect to each parameter can be derived exactly by backpropagation, and the minimization of the cost function can be done using simple stochastic gradient descent. In contrast, the gradient with respect to each parameter in RBM must be approximated by sampling from Markov chains [22–27]. Experiments have shown that NADE often outperforms other models in the task of estimating distribution, while the performance of NADE in some other tasks such as the unsupervised learning of features and initialization of neural networks is not so excellent. In this paper, we mainly deal with these two problems.

## 3. A Reweighted Scheme for Features

The features are totally determinated by the learned weight **W** and the bias **c** wherever in RBM or NADE. To improve the features, we try to modify the corresponding parameters learned by the model while keeping the structure of NADE.

A direct idea is to take advantage of the conditional probability computed by NADE. Consider the probability of one dimension of the input conditioned on the other dimensions; to measure the importance of the specified dimension, we clamp the states of the other dimensions and simply compare the probabilities of two cases as follows:

(7) $$\begin{array}{c} \frac{p\left(v_{i}=1,v_{\neq i}\right)}{p\left(v_{i}=0,v_{\neq i}\right)}=\frac{p\left(v_{i}=1\left|v_{\neq i}\right.\right)\cdot p\left(v_{\neq i}\right)}{p\left(v_{i}=0\left|v_{\neq i}\right.\right)\cdot p\left(v_{\neq i}\right)}=\frac{p\left(v_{i}=1\left|v_{\neq i}\right.\right)}{p\left(v_{i}=0\left|v_{\neq i}\right.\right)}=\omega_{i}. \end{array}$$

In this case, we define *ω*_*i*_ as the weight score for the *i*-th dimension of the input. Large or small value of *ω*_*i*_ indicates that the probabilities of these two cases vary drastically, and we should pay more attention to this specified dimension. This reweighted scheme never works in practice because of the huge amount of computation. For each dimension of every observation, we must compute two feed-forward procedures and it is impractical.

To deal with the problem, we approximate the conditional probabilities *p*(*v*_*i*_=1|**v**_≠*i*_) and *p*(*v*_*i*_=0|**v**_≠*i*_) by the fixed-order conditional probabilities *p*(*v*_*i*_=1|**v**_<*i*_) and *p*(*v*_*i*_=0|**v**_<*i*_), which is compatible with the original structure of NADE. This approximation drastically reduces the cost of computation by a factor of *H*, which is the size of each hidden layer.

We further replace *ω*_*i*_=(*p*(*v*_*i*_=1|**v**_<*i*_))/(*p*(*v*_*i*_=0|**v**_<*i*_)) by *ω*_*i*_=|*p*(*v*_*i*_=1|**v**_<*i*_) − *p*(*v*_*i*_=0|**v**_<*i*_)| to control the instability of *ω*_*i*_. Thus, we use each *ω*_*i*_ to modify the corresponding weight in the matrix **W**, in other words, the *i*-th column of **W**. We may pay more attention to the dimensions in which the probabilities change intensely in two different cases. These dimensions should have larger weights to generate the feature representation.

The final reweighted scheme is represented as

(8) $$\begin{array}{c} \omega_{i}=\left|p\left(v_{i}=1\left|v_{<i}\right.\right)-p\left(v_{i}=0\left|v_{<i}\right.\right)\right|, \end{array}$$

(9) $$\begin{array}{c} {\tilde{\omega }}_{i}=\left\{\begin{array}{cc} \omega_{i}, & \omega_{i}<\tau ,\\ \tau , & \text{otherwise,} \end{array}\right. \end{array}$$

(10) $$\begin{array}{c} k=\overset{D}{\underset{i}{\sum }}{\tilde{\omega }}_{i}, \end{array}$$

(11) $$\begin{array}{c} {\hat{\omega }}_{i}=\frac{D{\tilde{\omega }}_{i}}{k}, \end{array}$$

(12) $$\begin{array}{c} h=\text{sig}\left(c+\overset{D}{\underset{i}{\sum }}{\hat{\omega }}_{i}W_{\cdot ,i}v_{i}\right), \end{array}$$

where *τ* ∈ [0,1] is the threshold to control the difference, *D* is the size of input, ${\hat{\omega }}_{i}$ is the weight score of the *i*-th dimension, **W**_·,*i*_ is the *i*-th column of **W**, and **h** is the final reweighted feature.

In the reweighted procedure, equation (8) computes the difference between the probabilities in two cases for each dimension. Equation (9) controls the scale of the weight. Equations (10) and (11) normalize the weight.

While this reweighted scheme seems plausible, it seldom improves the features. It may be explained that the reweighted score does change the activation in each dimension of the feature **h**, while it does not change the relative magnitude of the activations, which may be more important for a better representation.

In order to resolve this problem, we prefer to deal with the rows of **W** rather than columns, and we would again utilize the structure provided by NADE. For each dimension of the input, the NADE provides a corresponding hidden layer, which could be used to modify the learned features. In this case, we pay more attention to the dimensions of the hidden layers which are over saturated or inactivated. These ideas lead to the following new reweighted scheme:

(13) $$\begin{array}{c} \tilde{h}=\frac{\sum_{i}^{D}h_{i}}{D}, \end{array}$$

(14) $$\begin{array}{c} {\tilde{\omega }}_{j}=\left\{\begin{array}{cc} \varepsilon_{\text{upper}}, & {\tilde{h}}_{j}>\tau_{\text{upper}},\\ \varepsilon_{\text{lower}}, & {\tilde{h}}_{j}<\tau_{\text{lower}},\\ 1, & \text{otherwise,} \end{array}\right. \end{array}$$

(15) $$\begin{array}{c} k=\overset{H}{\underset{i}{\sum }}{\tilde{\omega }}_{j}, \end{array}$$

(16) $$\begin{array}{c} {\hat{\omega }}_{j}=\frac{H{\tilde{\omega }}_{j}}{k}, \end{array}$$

(17) $$\begin{array}{c} {\hat{c}}_{j}=c_{j}\cdot {\hat{\omega }}_{j}, \end{array}$$

(18) $$\begin{array}{c} {\hat{W}}_{j,\cdot }={\hat{\omega }}_{j}W_{j,\cdot }, \end{array}$$

(19) $$\begin{array}{c} h=\mathrm{sig}\left(\hat{c}+\overset{D}{\underset{i}{\sum }}{\hat{W}}_{\cdot ,i}v_{i}\right), \end{array}$$

where *D* is the size of input, **h**_*i*_ is the *i*-th hidden layer in NADE, {*ε*_upper_, *ε*_lower_} are the relative weights, {*τ*_upper_, *τ*_lower_} are thresholds which control the value of activation, ${\tilde{h}}_{j}$ is the *j*-th unit in the normalized hidden layer $\tilde{h}$, and ${\hat{W}}_{j,\cdot }\text{ and}\;\;W_{j,\cdot }$ represent the *j*-th row for each matrix.

We conclude the reweighted procedure in Algorithm 1.

This reweighted scheme for features deserves explanation a bit more. As the activations of each hidden layer form a corresponding vector of the same size, in step one, we sum the vectors and normalize it to obtain the result $\tilde{h}$. Thus, $\tilde{h}$ is the average value of the activations for each dimension of the hidden layer, and it is a measure for how activated the dimension is during the feed-forward procedure in NADE.

We then introduce two thresholds {*τ*_upper_, *τ*_lower_} to control the activations. The unit is considered to be over saturated if the activation is larger than the upper threshold *τ*_upper_, and the corresponding dimension of this unit is endowed with a weight *ε*_upper_. Similarly, we give a weight *ε*_lower_ to the dimensions where the activations are smaller than the lower threshold *τ*_lower_. It should be noted that the weight *ε*_lower_ and *ε*_upper_ are relative values compared with the standard value 1. In practice, the weight *ε*_upper_ should be smaller than 1 while *ε*_lower_ should be larger than 1. We emphasize the importance of this procedure. The over saturated unit often affects the performance, and via this step, we set a smaller weight to alleviate this situation. While units with activation values close to zero are considered to be inactivated, these units should be kept inactivated, and some other units may even become inactivated after reweight. In this case, **W**_*j*,·_**v**+*c*_*j*_ is negative, and a large weight for this dimension confirms the situation. According to our point of view, this procedure forces the sparsity of the representation, which often leads to better performance.

We assume that the original reweighted score for each dimension is just one and normalizes the reweighted score to keep it reasonable.

Here, we emphasize that our aim is to improve the features. When we meet the problem of estimating distribution, the original weight of NADE should be utilized since the original weight is the optimal result for the maximum likelihood cost function. Our scheme is unsuitable for density estimation.

## 4. A Reweighted Scheme for Initialization

The weight learned by RBM or NADE can be used to initialize the weight of another neural network, which is one of the advantages of this kind of models. The further neural network may be used in other tasks such as classification.

We have proposed the reweighted scheme for features, which is applicable for each observation, while this method is unsuitable for initialization.

To solve this problem, we compute the reweighted score for each sample in the training set and take the average of them to obtain a new reweighted score for the weight matrix and bias. This procedure can be represented as

(20) $$\begin{array}{c} \hat{\omega }=\frac{{\sum^{}}_{k=1}^{N}{\hat{\omega }}_{k}}{N}, \end{array}$$

(21) $$\begin{array}{c} {\hat{c}}_{j}=c_{j}\cdot {\hat{w}}_{j}, \end{array}$$

(22) $$\begin{array}{c} {\hat{W}}_{j,\cdot }={\hat{\omega }}_{j}W_{j,\cdot }, \end{array}$$

where *N* is the number of samples in the training set, ${\hat{\omega }}_{k}$ is the reweighted score vector corresponding to the *k*-th training sample.

The complete process is concluded in Algorithm 2.

## 5. Experiments

In this section, we show the experimental results on several binary datasets with the reweighted scheme for both features and initialization. For the training procedure of NADE, a fixed order of dimensions of the input must be chosen in the beginning. Since experiments have shown that the ordering does not have a significant impact on the performance of NADE [11], for each dataset, the ordering is chosen independently and is kept the same during all the experiments on it. Furthermore, hyperparameters of NADE remain unchanged in order to select the hyperparameters of the reweighted scheme. Our implementation of the NADE model is based on the code provided by Larochelle and Murray [11].

### 5.1. Results on Learned Features

To test whether the reweighted scheme has improved the learned features, we perform some experiments on classification.

We note that our main purpose is to evaluate the proposed reweighted scheme rather than pursuing the best performance for classification, and we only use a moderate size of model to reduce the cost of computation. For each dataset, we first train a NADE and use Algorithm 1 to obtain the improved features. This procedure is processed for all the samples in training set, validation set, and test set which results in all new three corresponding sets. We then train a neural network with single hidden layer as the classifier on the learned features. The performance is measured by the classification error rate on test set. We further experiment on the features without reweighted scheme to obtain a standard result for comparison. A RBM with the same size of NADE is also trained and the classification result is used as reference.

We experiment on twelve different datasets from the UCI repository: Adult, Binarized-MNIST, Connect-4, Convex, DNA, Mushrooms, Newsgroups, OCR-letters, RCV1, Rectangles, SVHN, and Web. We list the details about these datasets in Table 1. The experimental results are shown in Table 2. We have chosen the best result for reweighted scheme among the results corresponding to different hyperparameters. We find that the classification error for features with reweighted scheme is lower than the one without reweighted, which proves the improvement on the original features. Features from the reweighted scheme may be more meaningful.

To further verify our method, we replace the neural network classifier with SVM, RandomForest, and AdaBoost and perform additional experiments. These experiments are implemented via LIBSVM [28] and scikit-learn. The results are shown in Tables 3–5. During all the experiments, the parameters of the classifiers have been optimized by grid search and validation which would give the best performance. The features with our reweighted scheme again outperform the original ones, and it confirms the effectiveness of our method.

Experimental results for different weights {*ε*_upper_, *ε*_lower_} on OCR-letters dataset are shown in Table 6. In this series of experiments, we train a NADE on the dataset at first, the learning rate is set to 0.001, the decrease constant is set to 0, the size of hidden layer is 100, and we use tied weight in NADE. That is, we set **V**=**W** in equation (6). Next, we keep *τ*_upper_=0.73  and  *τ*_lower_=0.27 and only modify the reweighted parameters to explore the performance of the reweighted scheme. The results have demonstrated that the reweighted scheme has a decisive role in improving the features. An unreasonable reweighted scheme often leads to a worse result than the one without reweighted scheme. We have found that setting the lower weight *ε*_lower_ larger than 1 and the upper weight *ε*_upper_ smaller than 1 seems to be a reasonable reweighted scheme. In the previous sections, we have already explained this manner that a smaller value for upper weight makes the over saturated unit to be less saturated, which is beneficial for the representation, while a larger value for lower weight preserves the inactivated unit and forces the feature to be sparse.

It should also be noted that the weights {*ε*_upper_, *ε*_lower_} are relative weight compared with the standard weight 1. Thus, the weight must be controlled and a too large or too small weight leads to a terrible result.

Another factor which influences the performance of the reweighted scheme is the thresholds {*τ*_upper_, *τ*_lower_} that explicitly control the unit to be saturated or inactivated. Results for different thresholds {*τ*_upper_, *τ*_lower_} on OCR-letters dataset are shown in Table 7. As the same as what we have done before, we only modify these two thresholds during this series of experiments, and we set the upper weight to 0.6 and the lower weight to 1.4. Results have also demonstrated the importance of the thresholds. On the one hand, the upper threshold controls the proportion of units which are seen to be over saturated, and a larger value of the upper threshold leads to a smaller proportion of these units. On the other hand, the lower threshold controls the proportion of units which are seen to be inactivated, and a smaller value of the lower threshold leads to a smaller proportion of these units. These units would be even more inactivated after the reweighted scheme.

From our point of view, these two thresholds depend more on the dataset rather than the specification. Still, as a conservative strategy, we prefer to set the upper threshold in the range from 0.5 to 0.8, while the lower threshold in the range from 0.5 to 0.2.

**Figure 1:**
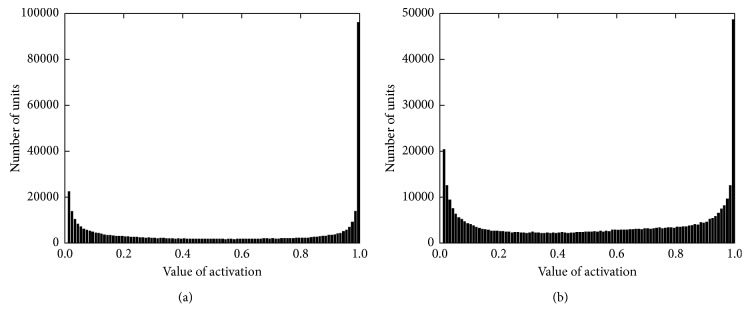
The number of units corresponding to each value of activations on OCR-letters. (a) Features before reweight. (b) Features after reweight.

To further investigate the features, we examine and analyze the value of activations of all the features in test set of OCR-letters.  Figure 1 shows the number of units corresponding to each value of activations from 0 to 1 with a step of 0.01. Units whose value of activation under 0.01 are ignored to keep the figure balance since these units make up a large majority of all units. The number of these units before reweight is 562453 and 575928 after reweight, which proves that the policy we proposed does keep the inactivated units and does even force the features more sparse. A significant decrease of the over saturated units is shown in figure, which accords with our purpose.

**Figure 2:**
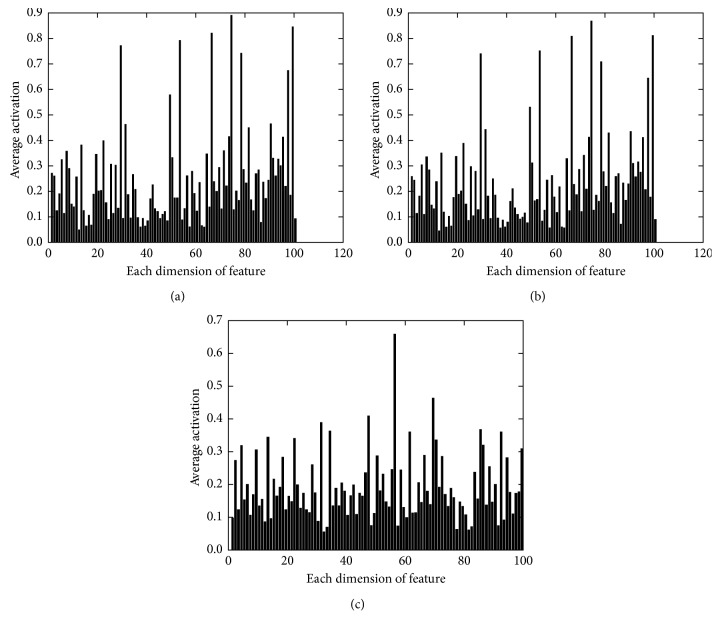
Average value of activations for each dimension on OCR-letters. (a) Features before reweight. (b) Features after reweight. (c) Features from RBM.

We also investigate the average value of activation for each dimension in the feature. The results are shown in Figure 2. We find that in the NADE features after reweight, the over saturated dimensions are restrained, while the inactivated dimensions are kept or even more inactivated. The average features before and after reweight are similar while the NADE features and RBM features vary dramatically. The difference between NADE features and RBM features is due to the intrinsic difference between the model NADE and RBM.

### 5.2. Results on Initialization

The reweighted scheme we have proposed also improves the performance of the neural network by initialization, which we would show here.

To test the performance, we train a NADE for each dataset, and a RBM with same size is also trained. We then use the learned weight matrix **W** and the bias **c** to initialize the parameters of the neural network classifier. Then, the neural network classifier is trained on the corresponding dataset. To evaluate our reweighted scheme, the parameters after reweight are also used as the initialization of another neural network classifier with same size. Finally, the performance is measured by the classification error.

We have shown the results in Table 8. As before, we perform experiments on the same twelve datasets and with the same hyperparameters. This time, from the results, we could see that the reweighted scheme for initialization has made a more significant improvement on the classification performance compared with the original NADE parameters. In most of the datasets, the difference between the errors of the reweighted-NADE and NADE is much larger than the one between NADE and RBM, which demonstrates the efficiency of the proposed reweighted scheme. In OCR-letters, the classification performance for reweighted-NADE is not as good as RBM, and this can be explained as the inherent difference between the parameters learned by NADE and RBM, which is hard to eliminate only via reweighted scheme. Anyhow, the proposed scheme always surpasses the one without reweighted.

In order to make the experiments more complete, we experiment on various weights {*ε*_upper_, *ε*_lower_} on the Web dataset and the results are shown in Table 9. For NADE, on this dataset, the learning rate is set to 0.005, the decrease constant is set to 0, the size of hidden layer is 150, and the weight is untied. Upper threshold and lower threshold are kept to 0.67 and 0.33. The heuristic reweighted method, which sets the lower weight larger than 1 and the upper weight smaller than 1, once again proved to be effective. While this time, the proper weights are more far away from the standard weight 1. This can be explained by the effect of the average. Since we compute the average of the reweighted score of all the training samples, a more discriminated reweighted scheme maintains the differences among the dimensions in the final reweighted score vector. In other words, we prefer a larger value for lower weight and a smaller value for upper weight when dealing with the problem of initialization.

Results about various thresholds on dataset Web are shown in Table 10. Upper weight and lower weight are set to 0.6 and 1.4, respectively. We make a similar conclusion to the one in previous section. The thresholds depend more on the dataset and we prefer a conservative strategy.

## 6. Conclusion

In this paper, we have proposed a simple and novel reweighted scheme to modify the learned parameters of NADE. We make use of the activations in hidden layers of the learned NADE model and set appropriate thresholds to control the proportions of the over saturated and inactivated units. In order to achieve better results, a heuristic reweighted method is proposed. The original parameters are modified and normalized. The reweighted parameters are used to generate better features or to improve the performance of the initialization for a neural network. The experiments have shown the effectiveness of the reweighted scheme, and there are evident improvements in both two important tasks in the field of machine learning.

## Figures and Tables

**Algorithm 1:**
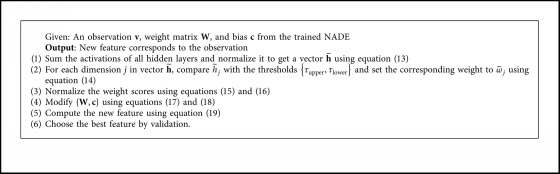
A reweighted scheme for new feature.

**Algorithm 2:**
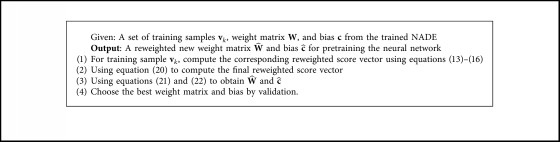
A reweighted scheme for initialization.

**Table 1:** Details about the twelve datasets.

Dataset	Input size	Number of classes	Training	Validation	Testing
Adult	123	2	5000	1414	26147
Binarized-MNIST	784	10	50000	10000	10000
Connect-4	126	3	16000	4000	47557
Convex	784	2	6000	2000	50000
DNA	180	3	1400	600	1186
Mushrooms	112	2	2000	500	5624
Newsgroups	5000	20	9578	1691	7505
OCR-letters	128	26	32152	10000	10000
RCV1	150	2	40000	10000	150000
Rectangles	784	2	1000	200	50000
SVHN	1024	11	594388	10000	26032
Web	300	2	14000	3188	32561

**Table 2:** Classification result via neural network on twelve datasets.

Dataset	Input size	Feature size	Size of NN	NADE	Reweighted-NADE	RBM
Adult	123	100	200	0.16074	**0.16013**	0.16598
Binarized-MNIST	784	300	400	0.0247	**0.0243**	0.0250
Connect-4	126	100	200	0.22436	**0.22143**	0.23367
Convex	784	300	400	0.27742	**0.26124**	0.29242
DNA	180	100	200	0.15683	**0.15008**	0.15093
Mushrooms	112	100	200	0.0082	**0.0059**	0.0066
Newsgroups	5000	1000	2000	0.30286	**0.26156**	0.30007
OCR-letters	128	100	200	0.1459	0.1409	**0.1361**
RCV1	150	100	200	0.05461	**0.04674**	0.06034
Rectangles	784	300	400	0.09566	**0.08782**	0.10204
SVHN	1024	300	400	0.08455	**0.08025**	0.09050
Web	300	150	200	0.02189	**0.02079**	0.02515

**Table 3:** Classification result via SVM on twelve datasets.

Dataset	NADE + SVM	Reweighted-NADE + SVM	RBM + SVM
Adult	0.15876	**0.15753**	0.16495
Binarized-MNIST	0.0374	**0.0349**	0.0358
Connect-4	0.27130	**0.26680**	0.28696
Convex	0.30036	**0.28570**	0.31466
DNA	0.13406	**0.12985**	0.14418
Mushrooms	0.01085	**0.00871**	0.00996
Newsgroups	0.29714	**0.26862**	0.28208
OCR-letters	0.1851	0.1802	**0.1692**
RCV1	0.06041	**0.05325**	0.07041
Rectangles	0.10836	**0.09864**	0.11526
SVHN	0.09346	**0.08839**	0.09842
Web	0.02110	**0.01969**	0.02420

**Table 4:** Classification result via Random Forest on twelve datasets.

Dataset	NADE + RF	Reweighted-NADE + RF	RBM + RF
Adult	0.16399	**0.16069**	0.16587
Binarized-MNIST	0.0301	**0.0265**	0.0312
Connect-4	0.26783	**0.25443**	0.28364
Convex	0.29632	**0.28502**	0.30658
DNA	0.16948	**0.15346**	0.15936
Mushrooms	0.01227	**0.01014**	0.01174
Newsgroups	0.32192	**0.29460**	0.31459
OCR-letters	0.1986	0.1863	**0.1708**
RCV1	0.07265	**0.06249**	0.08258
Rectangles	0.11816	**0.10624**	0.12646
SVHN	0.09838	**0.09339**	0.10283
Web	0.02647	**0.02368**	0.02773

**Table 5:** Classification result via AdaBoost on twelve datasets.

Dataset	NADE + AB	Reweighted-NADE + AB	RBM + AB
Adult	0.16013	**0.15677**	0.16445
Binarized-MNIST	0.0313	**0.0274**	0.0309
Connect-4	0.23912	**0.22737**	0.25494
Convex	0.29420	**0.28206**	0.30082
DNA	0.14250	**0.13238**	0.14587
Mushrooms	0.01049	**0.00853**	0.00978
Newsgroups	0.30753	**0.28115**	0.29474
OCR-letters	0.1681	0.1571	**0.1432**
RCV1	0.06847	**0.06072**	0.07509
Rectangles	0.10522	**0.09476**	0.11762
SVHN	0.08866	**0.08374**	0.09438
Web	0.02279	**0.02147**	0.02512

**Table 6:** Classification result for different weights on OCR-letters.

Upper-weight	Lower-weight	Error
0.8	0.8	0.1477
0.9	0.9	0.1472
1.0	1.0	0.1459
1.1	1.1	0.1445
1.2	1.2	0.1459
1.1	0.9	0.1470
1.2	0.8	0.1465
0.9	1.1	0.1447
0.8	1.2	0.1427
0.7	1.3	0.1425
0.6	1.4	**0.1411**

**Table 7:** Classification result for different thresholds on OCR-letters.

Upper-threshold	Lower-threshold	Error
0.55	0.45	0.1434
0.58	0.42	0.1434
0.61	0.39	0.1447
0.64	0.36	0.1431
0.67	0.33	**0.1409**
0.70	0.30	0.1424
0.73	0.27	0.1411
0.76	0.24	0.1418
0.79	0.21	0.1433

**Table 8:** Classification result for initialization on twelve datasets.

Dataset	Input size	Hidden layer size	NADE	Reweighted-NADE	RBM
Adult	123	100	0.15921	**0.15814**	0.16009
Binarized-MNIST	784	300	0.0216	**0.0198**	0.0205
Connect-4	126	100	0.19326	**0.18474**	0.19725
Convex	784	300	0.25672	**0.23898**	0.26394
DNA	126	100	0.06998	**0.05986**	0.06324
Mushrooms	112	100	0.00729	**0.00498**	0.00605
Newsgroups	5000	1000	0.34231	**0.28767**	0.3291
OCR-letters	128	100	0.1669	0.1601	**0.1374**
RCV1	150	100	0.05131	**0.04449**	0.05567
Rectangles	784	300	0.09062	**0.08338**	0.09948
SVHN	1024	300	0.08405	**0.07810**	0.08889
Web	300	150	0.01477	**0.01366**	0.01492

**Table 9:** Classification result with initialization for different weights on web.

Upper-weight	Lower weight	Error
0.8	0.8	0.01409
0.9	0.9	0.01477
1.0	1.0	0.01477
1.1	1.1	0.01480
1.2	1.2	0.01480
1.1	0.9	0.01446
1.2	0.8	0.01486
0.9	1.1	0.01477
0.8	1.2	0.01471
0.7	1.3	0.01468
0.6	1.4	0.01418
0.5	1.5	0.01455
0.4	1.6	0.01375
0.3	1.7	**0.01366**
0.2	1.8	0.01437

**Table 10:** Classification result with initialization for different thresholds on web.

Upper-threshold	Lower threshold	Error
0.55	0.45	0.01425
0.58	0.42	0.01434
0.61	0.39	0.01449
0.64	0.36	0.01446
0.67	0.33	**0.01418**
0.70	0.30	0.01431
0.73	0.27	0.01464
0.76	0.24	0.01446
0.79	0.21	0.01452

## Data Availability

All the datasets used in this paper are publicly available and could be obtained from http://archive.ics.uci.edu/ml/datasets.html.
